# Editorial: Diagnosis, treatment and prognosis of viral hepatitis, volume II

**DOI:** 10.3389/fmed.2022.1098625

**Published:** 2023-01-11

**Authors:** Jian Wu, Zhipeng Xu, Chuanlong Zhu, Wenyu Lin, Yijin Wang

**Affiliations:** ^1^Department of Clinical Laboratory, Gusu School, The Affiliated Suzhou Hospital of Nanjing Medical University, Suzhou Municipal Hospital, Nanjing Medical University, Suzhou, Jiangsu, China; ^2^Department of Pathogen Biology, Jiangsu Province Key Laboratory of Modern Pathogen Biology, Nanjing Medical University, Nanjing, China; ^3^Department of Infectious Disease, The First Affiliated Hospital of Nanjing Medical University, Nanjing, China; ^4^Liver Center, Harvard Medical School, Massachusetts General Hospital, Boston, MA, United States; ^5^School of Medicine, Southern University of Science and Technology, Shenzhen, China

**Keywords:** viral hepatitis, diagnosis, prognosis, treatment, host immune response

Currently, the global burden of viral hepatitis is mainly caused by five biologically unrelated hepatitis viruses: hepatitis A virus (HAV), hepatitis B virus (HBV), hepatitis C virus (HCV), hepatitis D virus (HDV) and hepatitis E virus (HEV) [(Xiang et al.), (1)]. They have different transmission routes and susceptible population (2, 3). Viral hepatitis is an important global public health problem with high morbidity and mortality rates that seriously endanger the health and quality of life of hundreds of millions of people (4). According to the statistics, 1.1 million died due to viral hepatitis in 2019. Hepatitis B virus and hepatitis C virus infections are responsible for the majority of viral hepatitis deaths. It was estimated that there were approximately 296 million people infected with hepatitis B and 58 million people infected with hepatitis C (5). In 2015, the United Nations adopted a resolution to combat viral hepatitis as part of the agenda to achieve the 2030 sustainable development goals. This was followed in 2016 by the development of the first global strategy to eliminate viral hepatitis (6). In light of this, it is necessary to call on governments, parties and the population to strengthen the prevention, diagnose, and treatment of viral hepatitis in the control of viral hepatitis.

The early diagnosis and prognosis evaluation of viral hepatitis are of great significance for effective treatment. Nevertheless, globally, < 5% of population with chronic viral hepatitis are aware of their condition (7). On account of the lack of awareness of viral hepatitis infection, antiviral treatment is often overlooked, which leads to the progression of the disease. Hence, it is of great significance to enhance patients' awareness of the harm of viral hepatitis, to diagnose viral hepatitis infection timely and accurately, to better estimate the prognosis of patients, and to find out new anti-viral treatment. So the appearance of this special issue is very opportune. The purpose of this Research Topic is to gather articles or reviews that contribute to a better understanding of viral hepatitis and provide new perspective and scientific theoretical grounding for the diagnosis and treatment of viral hepatitis.

To explore the markers or factors related to the occurrence, development and prognosis of viral hepatitis is conducive to the early prevention and treatment of the disease. In this special issue, Chen et al. reported that serum sTim-3 level was increased in patients with HBV, HCV, or HEV infection and gradually elevated in patients with either hepatitis or hepatitis with hepatic fibrosis. The roles of immune checkpoint molecules expressed on CD4 + T cells in chronic asymptomatic HBV carriers (ASCs) with HBeAg-negative were confirmed by Cui et al.. Yu et al. also revealed the associations between sustained low-level expression of HBsAg and mutated S gene sequence characteristics, protein function changes, and HBsAg immune complex formation in ASCs. Yang et al. identified the associations of Fc receptor-like 5 gene polymorphisms and mRNA expression levels with liver fibrosis in CHB patients. Wang et al. revealed that serum pregenomic RNA and hepatitis B core-related antigen were conductive to the prediction of the risk of virological recurrence in CHB patients after discontinuation of nucleos(t)ide analogs. Through the prospective multicenter observational cohorts, Zhu et al. reported the prevalence and adverse consequence of HBV reactivation in CHB patients with acute exacerbations. In additions, Xu et al. suggested the differentiated impact of the different types of acute decompensation events on the subsequent risk of nosocomial infections.

At present, the treatment of viral hepatitis is also one of the key concerns of researchers. Chuang et al. designed a protease-activatable retention probe for tracking HCV NS3/4A protease activity and distribution *via* positron emission topography imaging, which could optimize the protease-based therapies. Through the real-world experience, Peng et al. observed that PEG-IFNα2b add-on therapy was related to elevated eGFR in patients with CHB who received entecavir therapy. Salpini et al. conducted a review that showed that hepatitis B virus DNA integration is a new biomarker of HBV-mediated pathogenetic properties and an obstacle to current HBV therapeutic strategies. Meanwhile, Zhu et al. reviewed current treatment of chronic hepatitis B from clinical aspects.

It is also of great significance to state the relationship between hepatitis and other diseases and its occurrence in special populations, which can deepen people's understanding of viral hepatitis. Chen et al. reported the relationship between hepatitis C and kidney stone in US females. Chen et al. first reported the case of a man with very severe aplastic anemia who developed chronic hepatitis E after hematopoietic stem cell transplantation. Through a meta-analysis, Liu et al. indicated the prevalence of HBV in Chinese pregnant women. Finally, Zhao et al. explained the feasibility of eliminating hepatitis C in China from the perspectives of epidemiology, natural history and intervention.

The purpose of this Research Topic is to provide new perspective and scientific theoretical basis for the diagnosis, treatment and prognosis of various viral hepatitis.

## Author contributions

JW and YW drafted and critically revised the work. All authors had the idea for the article.
